# Factors affecting upper limb ergonomics in robotic colorectal surgery

**DOI:** 10.1093/jscr/rjad632

**Published:** 2023-11-20

**Authors:** Shing Wai Wong, Zhen Hao Ang, Ranah Lim, Xiuling Jasmine Wong, Philip Crowe

**Affiliations:** Department of General Surgery, Prince of Wales Hospital, Sydney, New South Wales, 2031, Australia; Randwick Campus, School of Clinical Medicine, The University of New South Wales, Sydney, New South Wales, 2052, Australia; Department of General Surgery, Prince of Wales Hospital, Sydney, New South Wales, 2031, Australia; Randwick Campus, School of Clinical Medicine, The University of New South Wales, Sydney, New South Wales, 2052, Australia; Department of General Surgery, Prince of Wales Hospital, Sydney, New South Wales, 2031, Australia; Department of General Surgery, Prince of Wales Hospital, Sydney, New South Wales, 2031, Australia; Department of General Surgery, Prince of Wales Hospital, Sydney, New South Wales, 2031, Australia; Randwick Campus, School of Clinical Medicine, The University of New South Wales, Sydney, New South Wales, 2052, Australia

**Keywords:** ergonomics, upper limb disorders, robotic colorectal surgery

## Abstract

The aim of the study was to examine the factors which may influence suboptimal ergonomic surgeon hand positioning during robotic colorectal surgery (RCS). An observational study of 11 consecutive RCS cases from June 2022 to August 2022 was performed. Continuous video footage of RCS cases was analysed concurrently with video recordings of surgeon’s hand positions at the console. The outcome studied was the frequency with which either hand remained in a suboptimal ergonomic position outside the predetermined double box outlines, as marked on the surgeon’s video, for >1 min. Situations which resulted in poor upper limb ergonomics were dissection in the peripheral operating field location, left-hand use, use of the stapler, dissection of the main mesenteric blood vessels, and multi-quadrant surgery. Being aware of situations when suboptimal ergonomic positions occur can allow surgeons to consciously compensate by using the clutch or pausing to take a rest break.

**What does this paper add to the literature?:**

The study is important because it is the first to look at factors which may influence poor upper limb ergonomics during non-simulated RCS. By recognizing these factors and compensating for them, it may improve surgeon ergonomics with resultant better performance.

## Introduction

The term ergonomics is derived from the Greek words ergon (labour) and nomia (arrangement) [1]. Ergonomics is the study of workplace design with the goal of optimizing technology to improve the interaction between surgeon and machine [2]. Studies have reported increasing levels of occupational injury related to laparoscopic and open surgery [3]. Improved ergonomics for the operating surgeon may be an advantage of robotic colorectal surgery (RCS) [4]. The most common cause of improper upper limb positioning during RCS occurs when the shoulders are abducted and the surgeons’ elbows are lifted off the armrest [5]. This can be avoided by using the clutch control system to reposition the control manipulators back to a neutral position [6]. Frequent judicious clutching can return the forearms to the neutral position with the elbows at 90°, tucked in, adjacent to the torso, and resting comfortably on the armrest [2, 7, 8].

Despite the upper limb ergonomic benefits, the use of the clutch can lead to a brain sensory misalignment of the location of surgeon hands at the console and the location of the robotic arms as visualized through the console binoculars [9]. Other researchers have shown that use of the clutch control is more frequent with initial coaching and increasing robotic experience [10, 11]. Experience may compensate for the increase cognitive load of visual-perception mismatch by frequent subconscious use of the clutch control to separate a long movement into shorter ones.

The aim of the study is to examine factors which may influence suboptimal ergonomic positioning of the surgeon’s hands outside of the central console position. Identifying these factors may allow surgeons to recognize situations when upper limb ergonomics may be compromised and consciously compensate for them by using the clutch control.

## Materials and methods

The robotic surgeries for colorectal disease were performed by a single surgeon (SW) with the da Vinci Xi system (Intuitive Surgical, Inc., Sunnyvale, CA, USA) at the Prince of Wales Private Hospital (Sydney, Australia). The surgeon was right-handed and had completed over 100 robotic colorectal cases prior to this study. The four robotic ports were placed obliquely in a straight-line, spaced 6–10 cm apart, as recommended by the manufacturer. For both right and left colonic resections, two left arms and one right arm configuration was the preferred set-up. In most cases, the two robotic arms adjacent to the camera-holding robotic arm were used for dissection.

**Figure 1:**
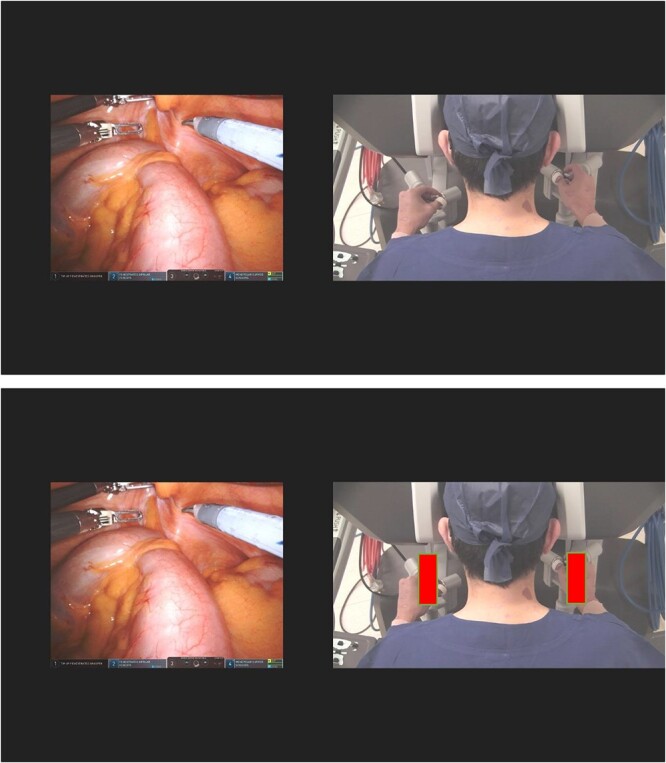
Concurrent videotaping of intraoperative case and surgeon at the robotic console (with central position outline marked in red).

This study followed the STROBE statement for cohort studies [12]. Ethics for this study was granted by the South Eastern Sydney Local Health District Health Research Ethics Committee, reference number: 2021/ETH11587. Continuous video footage of 11 consecutive RCS cases from June 2022 to August 2022 was analysed side by side with concurrent video recordings of the surgeon sitting at the robot console (Fig. 1). The video recording was performed with the robot and the operating theatre overhead light cameras. The patients consented to participate in the study.

Two investigators independently analysed the videos. Their findings were compared, and any discrepancies were discussed until a consensus was reached. The variables studied were morning or afternoon start time, operating time blocks (divided into 30-min blocks), operation type, task performed, anatomic target, instrument used, and hand involved. Use of the right and left clutch controls automated performance metrics were provided by Intuitive Surgical Inc. The outcome studied was the number of times either hand was outside the predetermined outlines marked on the surgeon/ robot console video for >1 min. The influence of variables on situations when the surgeon had not clutched the robotic controls into a more central ergonomic position was studied.

## Results

The mean age (and standard deviation) of the patients was 70 (19) (Table 1). There were six female patients and five male patients. The mean BMI was 25 (5). Six patients had BMI <25. All patients had total RCS without open conversion. The mean robotic console time was 172 min (54). For all cases, the mean number of clutch activations was 89 (25) on the left side and 77 (26) on the right side. For right-sided bowel resections, the mean was 103 (21) and 77 (17). For left-sided bowel resections, the mean was 82 (23) and 77 (30).

**Table 1:** Patient demographics.

Patient number	1	2	3	4	5	6	7	8	9	10	11
Age	81	85	62	33	86	40	81	89	83	49	81
Gender	M	M	F	F	F	M	F	M	F	F	M
BMI	25	28	29	23	28	18	19	22	36	30	22
Total console time (min)	129	165	198	193	194	287	106	189	103	112	216
Surgery type	RHC	RHC	eRHC	eRHC	LHC	LHC	rHart	HAR	HAR	HAR	ULAR
Indication	Ca	Ca	Ca	Pol	Ca	Ca	DD	Ca	Ca	DD	Ca
Suboptimal hand position >1 min	11	14	12	17	11	14	9	8	7	6	15
Suboptimal hand position >4 min	4	7	9	6	9	12	4	3	3	3	4
Right hand right		3		1	4	2	1	1	1	3	
Both hands right		2	10	1	3	5	2	6		3	1
Left hand left	6	1	1	9	1	4	5	1	4		7
Both hands left	5	6	1	4	1	3	1		2		7
Right hand right and left hand left		2		2							
Use of left clutch	79	136	105	92	104	122	51	83	77	55	79
Use of right clutch	57	100	85	65	116	127	47	72	74	45	57

RHC = right hemicolectomy; eRHC = extended right hemicolectomy; LHC = left hemicolectomy; rHart = reversal Hartmann; HAR = high anterior resection; ULAR = ultralow anterior resection; Ca = cancer; Pol = polyp; DD = diverticular disease

**Figure 2:**
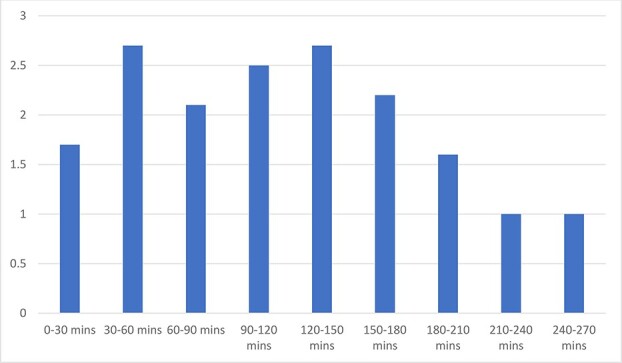
Average suboptimal ergonomic hand positions and 30-min operating time blocks.

The mean number of times the surgeon hands were in suboptimal ergonomic positions for >1-min duration per case was 11. The mean number of suboptimal ergonomic hand positions for >4-min duration was six. Five procedures were performed in the morning and six procedures were performed in the afternoon. The mean number of >1 min and 4 min suboptimal ergonomic hand positions was the same in both morning and afternoon groups (11 and 6, respectively). The mean number of suboptimal ergonomic hand positions was similar in each 30-min block of robotic console surgery time (Fig. 2). There was no significant difference in the total number of suboptimal ergonomic hand positions in relation to gender (62 for 6 females and 62 for 5 males) and to BMI (74 for 6 patients with BMI <25 and 50 for 5 patients with BMI >25).

**Figure 3:**
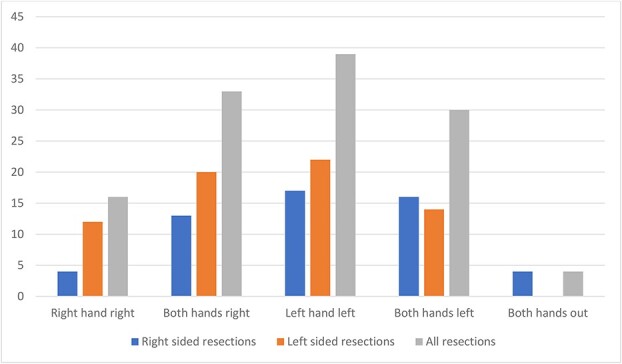
Pattern of suboptimal ergonomic hand positions.

The pattern of suboptimal ergonomic hand positions was evenly distributed apart from less isolated right hand suboptimal positioning (i.e. isolated right hand out to the right side) (Fig. 3). This pattern did not differ when comparing right- and left-sided bowel resections. Suboptimal ergonomic positioning of the hands occurred more commonly at the peripheral dissection sites, defined as the more lateral bowel section of the colorectal dissection on each side. For right hemicolectomy surgery, the total number of suboptimal positions was 35 for the peripheral sites (caecum and transverse colon) compared with 18 for the central sites (ascending colon and hepatic flexure). For left hemicolectomy surgery, the corresponding numbers were 19 (for peripheral transverse colon and descending colon sites) and 4 (for central splenic flexure site). For anterior resection/ reversal Hartmann surgery, the numbers were 19 (for descending colon and rectum) and 13 (for sigmoid colon). The total numbers for peripheral and central dissection were 73 and 35. Suboptimal ergonomic hand positions occurred a total of 17 times during dissection of the main mesenteric vessels (ileocolic and inferior mesenteric vessels) and a total of 4 times during dissection of the mesorectum (among the four rectal dissection cases). During situations of non-ergonomic hand position, the right-hand instrument being used was scissors 61 times, vessel sealer 44 times, stapler 9 times, suture holder 6 times, and sucker twice.

## Discussion

The study confirmed a preference for the surgeon to maintain his hands in an ergonomic position with the forearms supported on the console bar during RCS. The detrimental nature of our outcome factor of prolonged surgeon hand position outside the central console has been supported by other studies. Frequent clutching of the control manipulators can help maintain the elbows in a neutral position, avoid sustained shoulder abduction, and reduce tension of the intrinsic hand muscles [7]. Support for these optimal joint angles has been derived from the validated Rapid Upper Limb Assessment tool [13]. Supporting the weight of the arm reduces the shoulder posture score. Resting of the forearms on the armrest can reduce fatigue and improve the precision of forearm movements. The armrest load has been shown to be significantly higher and the workspace range to be significantly lower in experts compared with novices during simulated robotic exercises [8]. Studies have shown that uninterrupted periods of low-level activity to be associated with the development of musculoskeletal pain [14].

Surgeon cognitive workload has been reported to be lower on the robotic system compared with the laparoscopic system, which may lead to improved surgical performance [15–17]. Long operative times contribute to surgeon mental, physical, and visual fatigue [18, 19]. Fatigue typically sets in when surgical time approaches 4 h with symptoms including mental exhaustion, irritability, and impaired surgical judgement [20]. One study involving 15 surgeons performing exercises on the robotic trainer found that after 2 h, a statistically significant deterioration in performance was observed and this continued to worsen until exercise termination 4 h later [21]. The effect of fatigue on overall surgical proficiency may result in more impairment of cognitive performance than psychomotor skills [21–23]. Surprisingly, we did not find fatigue to be an important variable influencing suboptimal ergonomic positioning of the upper limbs. In our study, there was no difference detected when comparing morning and afternoon starting times and when comparing the different 30-min operating time blocks. It may be related to the ability to take periodic rest breaks as required [23]. Microbreaks with targeted stretching can interrupt long-lasting periods of low-level intensity and has been shown to reduce postprocedure musculoskeletal pain [24].

In our study, we found that suboptimal ergonomic hand positions occurred more frequently when operating at the most peripheral working space, with a ratio of more than two to one. This was consistent with the findings of Gabrielson *et al*. [6] This may be related to attempts to maintain a wider manipulation angle. The manipulation angle between two instruments during RCS has been shown to be greatest when the target was most central to a line perpendicular to the midpoint of a line joining the two active ports [25]. The surgeon may unconsciously separate their hands to compensate for the narrow manipulation angles associated with more distant targets.

In our study, suboptimal ergonomic positioning was frequent when dissecting the mesenteric vessels and may be related to separation of surgeon hands when dissecting tissue closer to the ports. Intense concentration during a task can distract the surgeon from clutching the controls to more ergonomic positions—e.g. during intracorporeal suturing. Manipulation of instruments with a long internal length such as the robotic stapler was expectedly associated with more frequent suboptimal ergonomic positioning.

Dalager *et al*. reported that the muscle activity in forearm and shoulder muscles was significantly greater on the right (dominant) side compared with the left for laparoscopic surgery but not for robotic surgery [26]. Robotic assistance has also been shown to improve the fine motor skills of the nondominant hand and confer virtual ambidexterity [27, 28]. The innate dominance of one hand’s dexterity over another may not be totally compensated for by the robot. One study reported correlation between handedness and surgical outcome with robotic prostatectomy [29]. Another study of robot-assisted radical prostatectomy found that surgeons with a lot of experience used their dominant hand more frequently compared with less experienced surgeons [30]. Similarly in our study, we found that isolated right hand to the right side suboptimal ergonomic position was half as frequent as the other three permutations and may be related to subconscious preference to maintain the dominant hand in a neutral position.

We anticipated more events with multi-quadrant colorectal surgery because of the need for wider range of surgeon hand movement compared with single-quadrant urological and gynaecological surgery. Single-quadrant surgery was associated with less frequent non-ergonomic positions (55 times involving 6 cases =9.1/ case) compared with multi-quadrant surgery (44 times involving 3 cases = 14.7/case). However, the difference may be explained by the average duration of the console time—134 min for single quadrant surgery and 202 min for multi-quadrant surgery.

There have been no previous study investigating factors which may influence poor upper limb ergonomics during non-simulated RCS. This observational study involved double analysis of side-by-side videos of consecutive RCS cases. Certain situations resulting in suboptimal upper limb ergonomics were able to be identified. There are limitations of our study. Despite analysis of the side-by-side videos by two independent observers, optimal interpretation and complete accuracy cannot be guaranteed. Only one operating surgeon was videotaped in this study which impacts on the generalizability of the findings. Physical and mental workloads were not evaluated in this study.

## Conclusion

In our study, we have identified factors which resulted in poor upper limb ergonomics during non-simulated RCS. Being aware of when suboptimal ergonomic positioning of the upper limbs occurs can allow the robotic surgeon to consciously compensate with use of the clutch or pause and take a rest break to reset physically and mentally.
